# Laparoscopic Heller Myotomy in a Patient With Achalasia and Isolated Situs Inversus of the Liver

**DOI:** 10.7759/cureus.60229

**Published:** 2024-05-13

**Authors:** Edward J Prange, Ziad Awad, Ruchir Puri

**Affiliations:** 1 Surgery, University of Florida College of Medicine, Jacksonville, USA; 2 Gastrointestinal Surgery, University of Florida College of Medicine, Jacksonville, USA

**Keywords:** esophagus, foregut, minimally invasive laparoscopy, minimally invasive, heller myotomy, isolated situs, dextrohepatica, abdominal situs inversus, visceral situs malposition, esophageal achalasia

## Abstract

Achalasia is a rare esophageal motility disorder characterized by incomplete lower esophageal sphincter (LES) relaxation, increased LES tone, and absent peristalsis in the esophagus. Management of achalasia includes pneumatic dilation (PD), Botulinum toxin A (BTA) injections to LES, per oral endoscopic myotomy (POEM), and a laparoscopic Heller myotomy (LHM). Situs inversus is a rare congenital condition in which the abdominal and thoracic organs are located in a mirror image of the normal position in the sagittal plane. We herein present a case of a patient with Type II achalasia who underwent an LHM and toupet fundoplication in the setting of an isolated laterality malposition of the liver on the left side of the abdomen. Single organ congenital lateralization defects are extremely rare with literature describing few case reports and case series. A much rarer condition is isolated organ situs inversus. In the foregut, most reports of isolated situs inversus are limited to isolated gastric situs inversus, dextrogastria. Most isolated liver malposition has described situs ambiguous, at the midline, usually associated with polysplenia. Our patient had the normal position of the foregut structures, including the stomach, spleen, pancreas, and duodenum, except for the isolated situs inversus of the liver. Because of the unusual anatomy, performing an LHM was quite challenging. Our workup approach and intraoperative considerations are described. By displacing the larger left lobe of the liver, we were able to safely complete a standard heller myotomy with adequate length and distally across the gastroesophageal junction. Our patient had an uncomplicated post-operative course, and at follow-up has continued to show improvements in her dysphagia and her quality of life.

## Introduction

Achalasia is a rare esophageal motility disorder characterized by incomplete lower esophageal sphincter (LES) relaxation, increased LES tone, and absent peristalsis in the esophagus. The prevalence appears to be around 10.82 per 100,000 people [1]. Achalasia is caused by progressive degeneration of ganglion cells in the myenteric plexus in the lower part of the esophagus resulting in progressive dysphagia, regurgitation, chest discomfort, and weight loss [1]. An upper endoscopy and esophagram can assist, but the cornerstone to establish the diagnosis is a high-resolution manometry (HRM) [2,3]. HRM typically shows aperistalsis of the esophagus and an increased LES pressure [2].^ ^Management of achalasia includes pneumatic dilation (PD), Botulinum toxin A (BTA) injections to LES, per oral endoscopic myotomy (POEM), and a laparoscopic Heller myotomy (LHM) [4,5].^ ^The response to these treatments varies based on patient factors and the type of achalasia diagnosed [4]. Situs inversus is a rare congenital condition in which the abdominal and thoracic organs are located in a mirror image of the normal position in the sagittal plane. The overall description of laterality-associated embryonic malposition is a spectrum from isolated organ involvement to situs inversus totalis. The overall incidence of laterality-associated defects is noted to be one person per 10,000 births [6]. The liver develops from the ventral foregut endoderm of the primitive gut, which starts at week six. The formation of the liver starts with a hepatic diverticulum. The anterior portion of the diverticulum gives rise to the liver and intra-hepatic biliary tree and the posterior part to the the gall bladder and extra-hepatic biliary tree. Malrotation of the liver is noted to be exceedingly rare with very few reports in literature. It is possible the origin of the hepatic diverticulum of the left side instead of the right leads to isolated malrotation of the liver. We herein present a case of a patient with achalasia who underwent an LHM in the setting of an isolated laterality malposition of the liver on the left side of the abdomen. All other organs were located in their normal anatomical positions with no polysplenia or asplenia present. Both these conditions are rare and reports of these coexisting are not currently present in the literature.

## Case presentation

**Figure 1:**
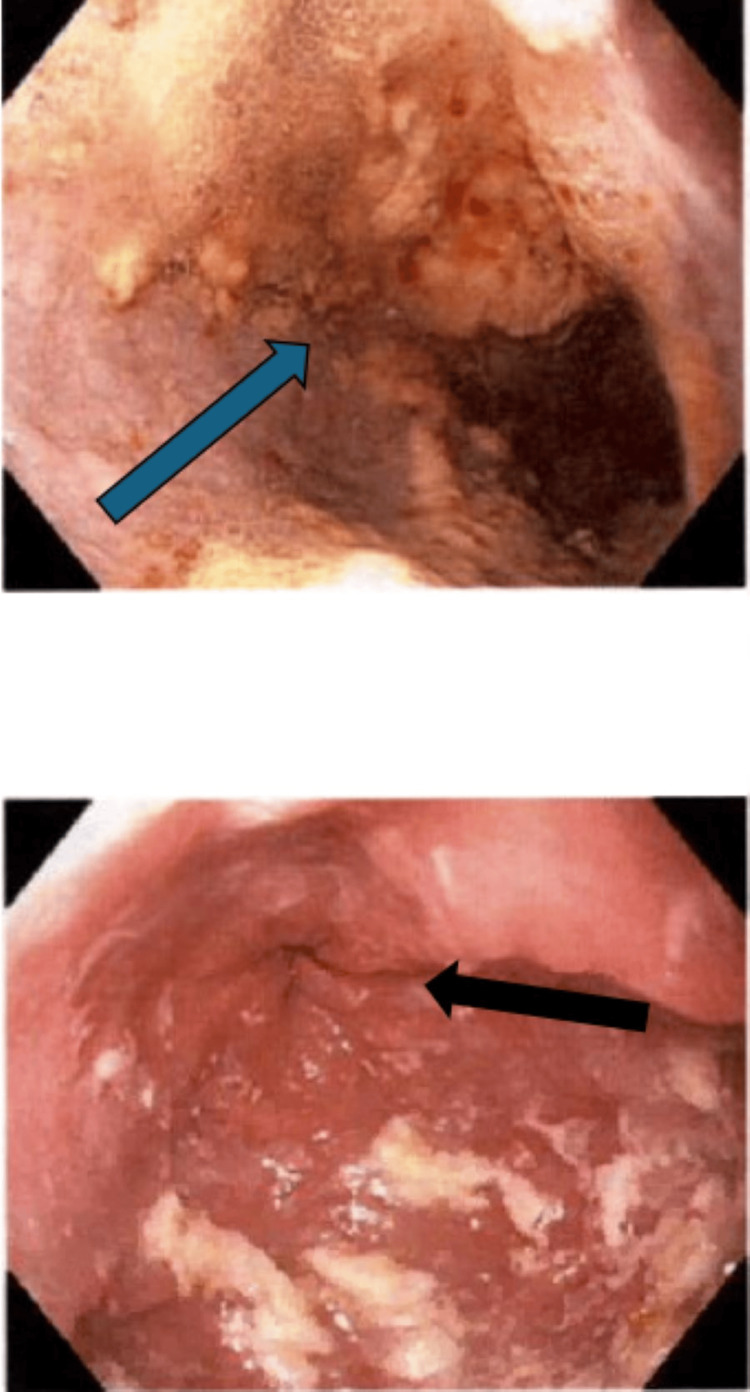
Dilated esophagus (above with blue arrow). Tight lower esophageal sphincter (below with black arrow)

**Figure 2:**
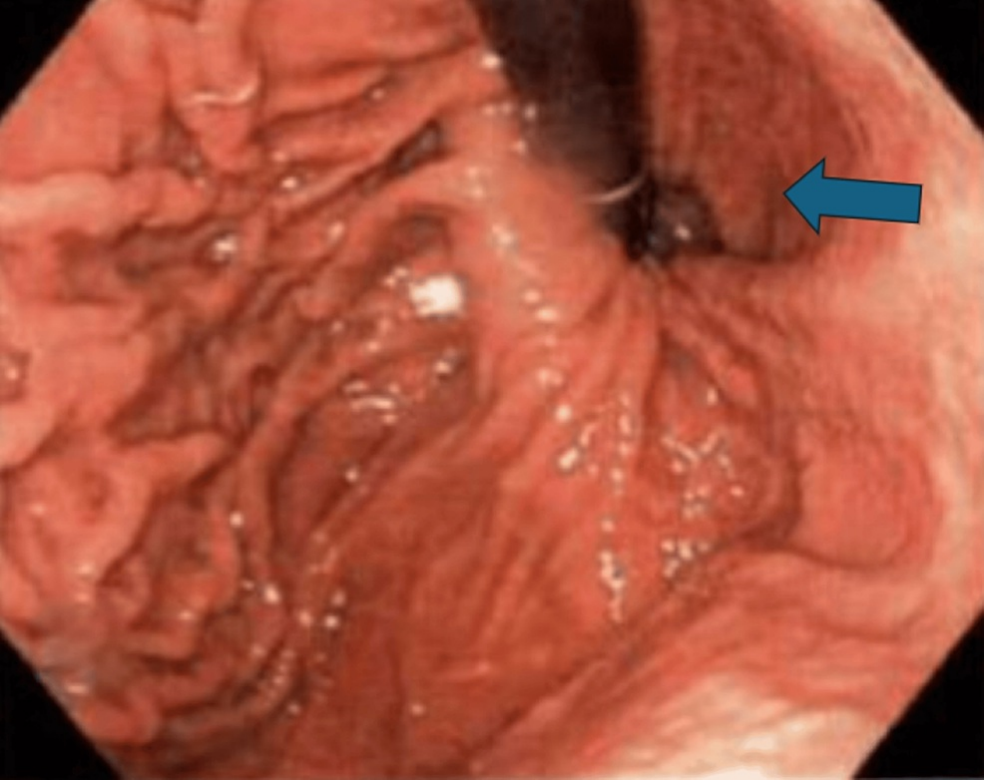
Hiatal hernia on retroflex (blue arrow)

**Figure 3:**
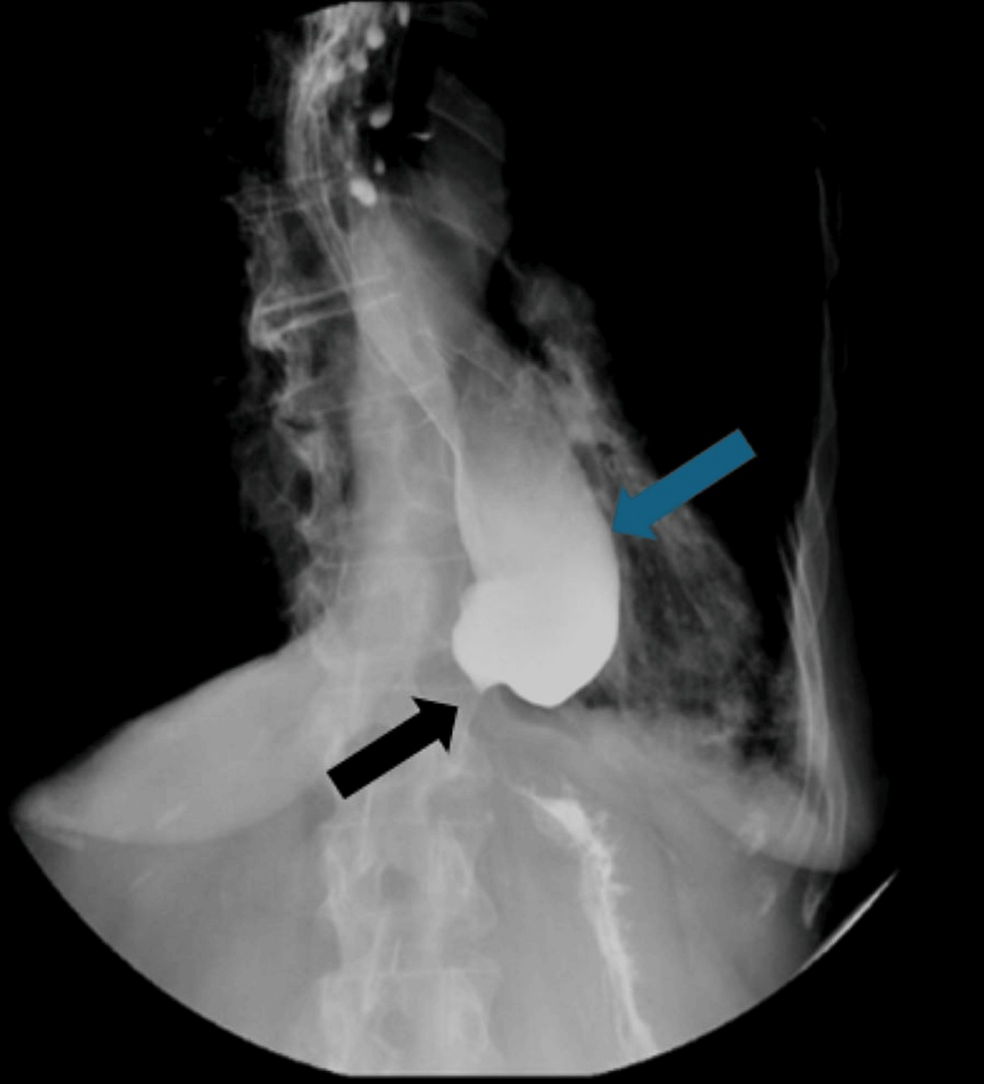
Esophagram showing dilated esophagus (blue arrow) and minimal to no flow through the gastroesophageal junction with bird's beak appearance (black arrow)

**Figure 4:**
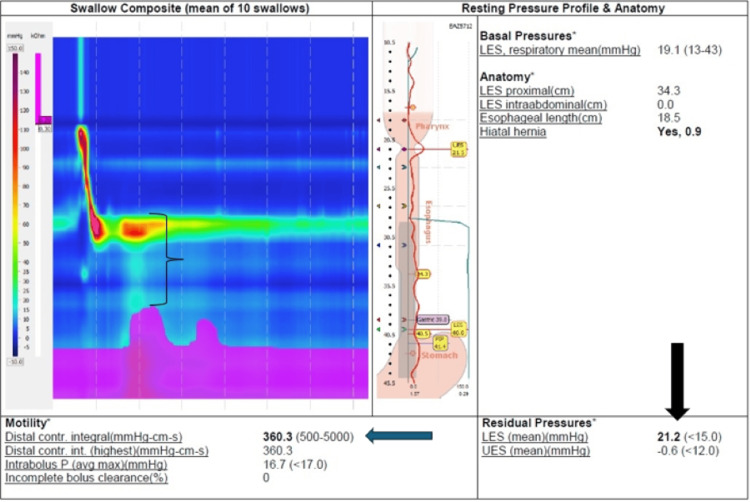
HRM consistent with type II achalasia; DCI of 360 (blue arrow). Pan esophageal pressurization (black bracket) and IRP of 21 (black arrow) HRM, high-resolution manometry; DCI, distal contraction integral; IRP, integrated relaxation pressure

**Figure 5:**
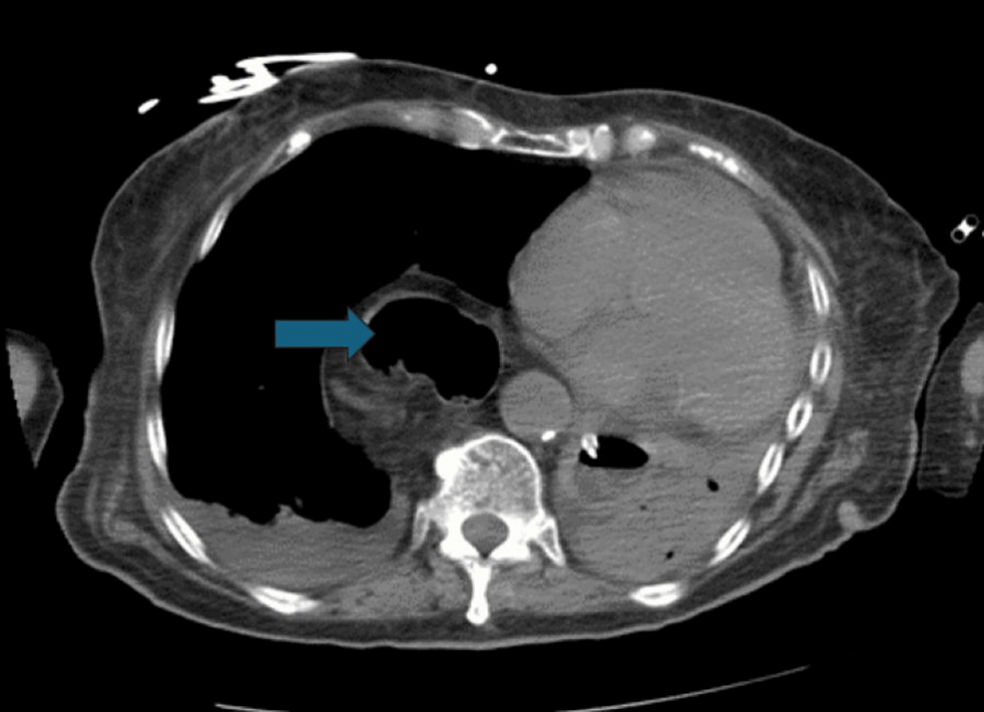
CT scan showing a dilated esophagus (blue arrow)

**Figure 6:**
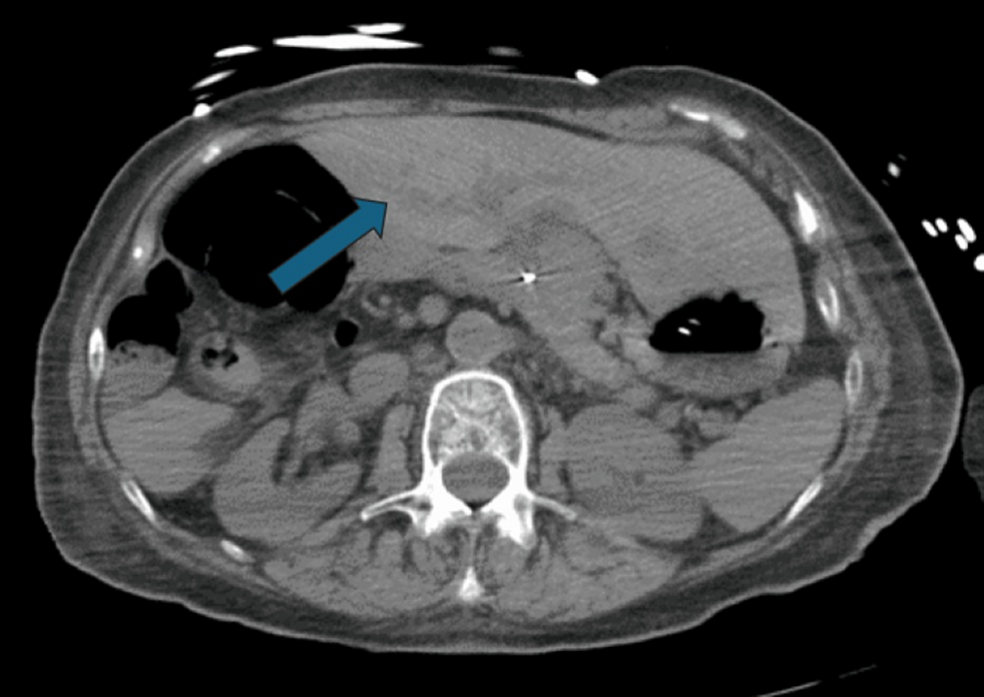
Isolated situs inversus of the liver (dextrohepatica) showing the left lobe of the liver on the right side of the patient (blue arrow)

A 74-year-old female with isolated situs inversus of the liver was referred to our hospital for investigation and treatment of type 2 achalasia. She reported that over the last eight to 10 months, she has been experiencing problems with food getting stuck in her throat and episodes of choking. She stated that she eats very little now and has to eat soft foods and soups to avoid choking, and she has had some weight loss. She reported weight loss of about 40 lb over the last 10 months and had a BMI of 17. She reported undergoing a PD three times in the last year. There were no other warning signs. Endoscopy showed a dilated esophagus with retained food and a non-malignant narrowing in the distal esophagus, along with a hiatal hernia (Figures 1, 2). The esophagram showed the typical bird’s beak appearance (Figure 3). Manometry revealed an integrated relaxation pressure (IRP) of 21, a lack of peristalsis, and pan esophageal pressurization of 90%, which was consistent with type 2 achalasia (Figure 4). CT Scan of the abdomen re-demonstrated a dilated esophagus and an isolated situs inversus of the liver: dextrohepatica (Figures 5, 6). We did not perform an MRCP to assess the liver anatomy. In hindsight, it would not have changed our management but would have provided additional information that potentially could have been beneficial. The CT scan shows an accurate image of what we found intra-operatively. 

**Figure 7:**
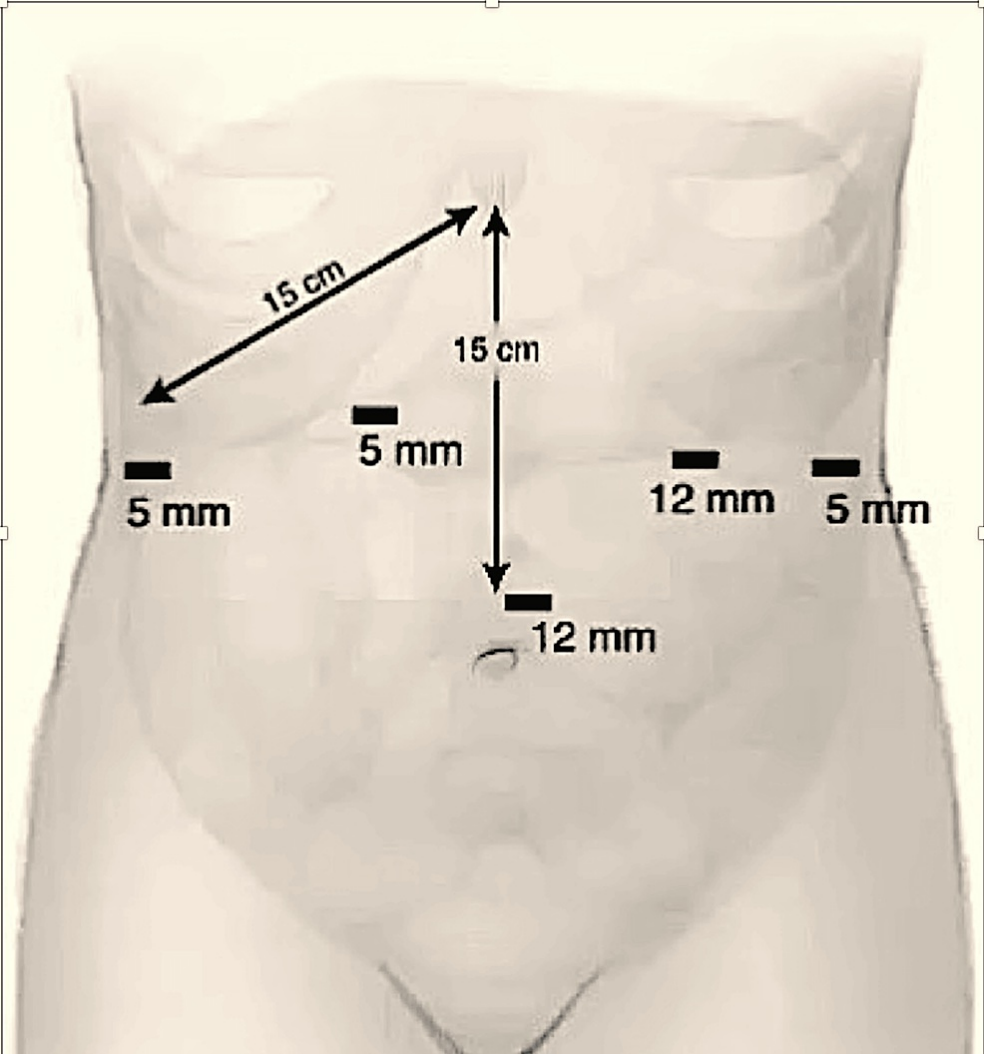
Trocar placement for a LHM showing the location and the size of the trocars LHM, laparoscopic Heller myotomy

**Figure 8:**
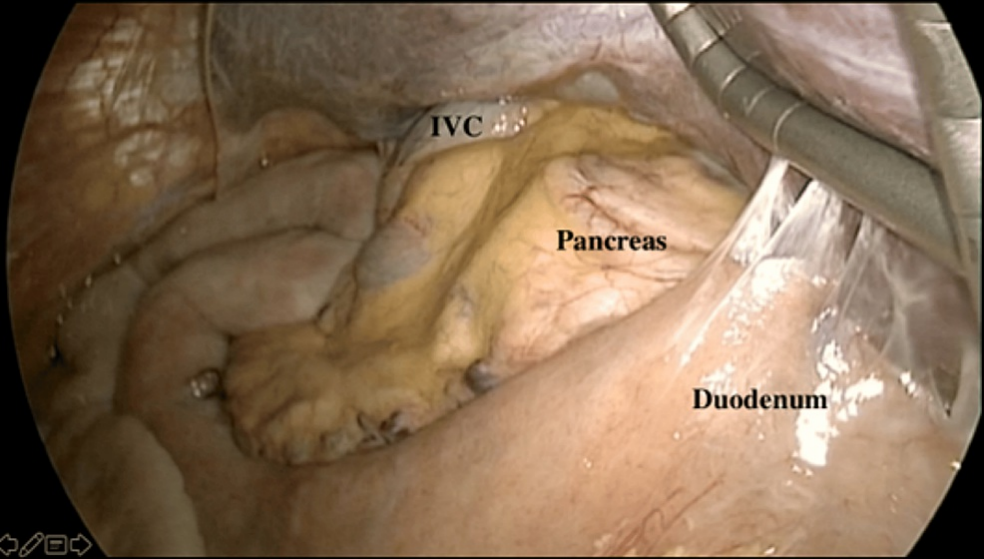
The left lobe of the liver to the patient's right side above the labeled IVC. Liver retractor under the right lobe on the patient's left side IVC, inferior vena cava

**Figure 9:**
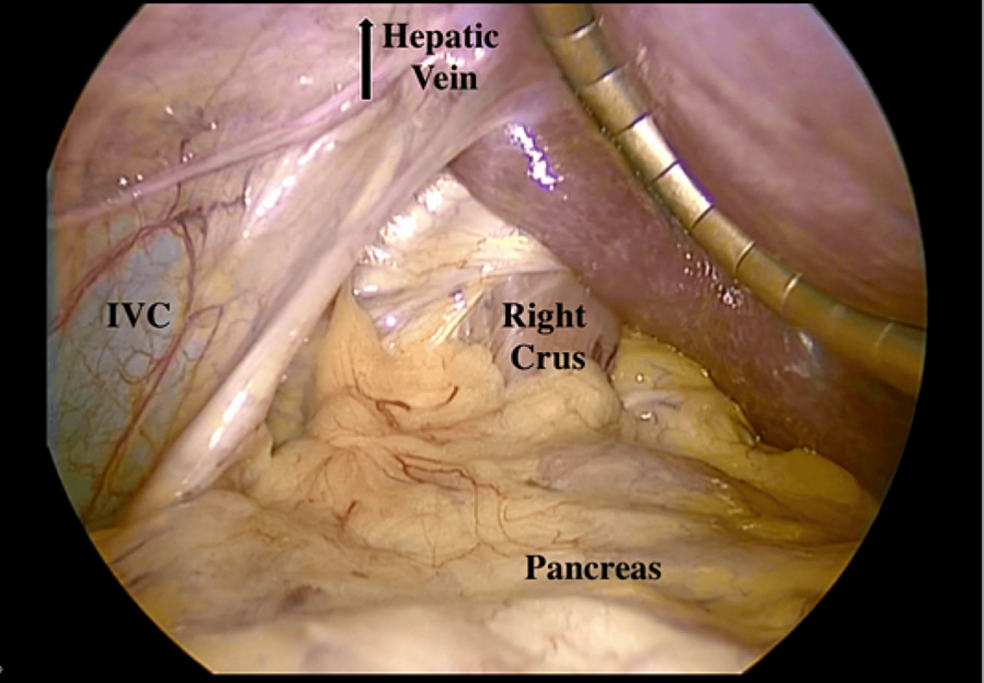
The right lobe of the liver on the patient's left side displaced by the liver retractor

After failed PDs, we did not consider BTA. We were concerned that it may not avoid an operation and the risk of esophageal injury in the setting of malrotation of the liver would increase after BTA. The operation was performed laparoscopically with the patient-placed supine, split leg and reverse Trendelenburg position. Standard five trocar placement for a foregut procedure was performed (Figure 7). A liver retractor was placed to retract the smaller right lobe of the liver (Figure 8) and allow for visualization of the surrounding viscera and vasculature. The inferior vena cava and hepatic veins were visualized with this unique anatomical variant. Dissection was carried out along the right crus (Figure 9), which was extremely challenging. After transection of the pars flaccida, the left crus was identified and hiatal dissection was performed. It was difficult to perform the mediastinal dissection and an appropriate angle for performing a safe and adequate esophageal myotomy was not clearly available.

**Figure 10:**
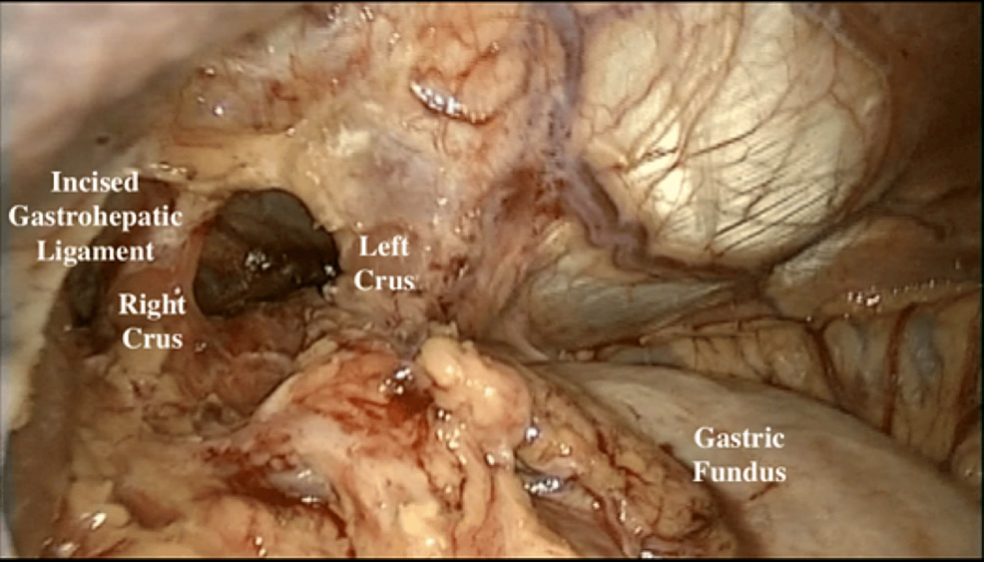
View of the hiatus after complete dissection

**Figure 11:**
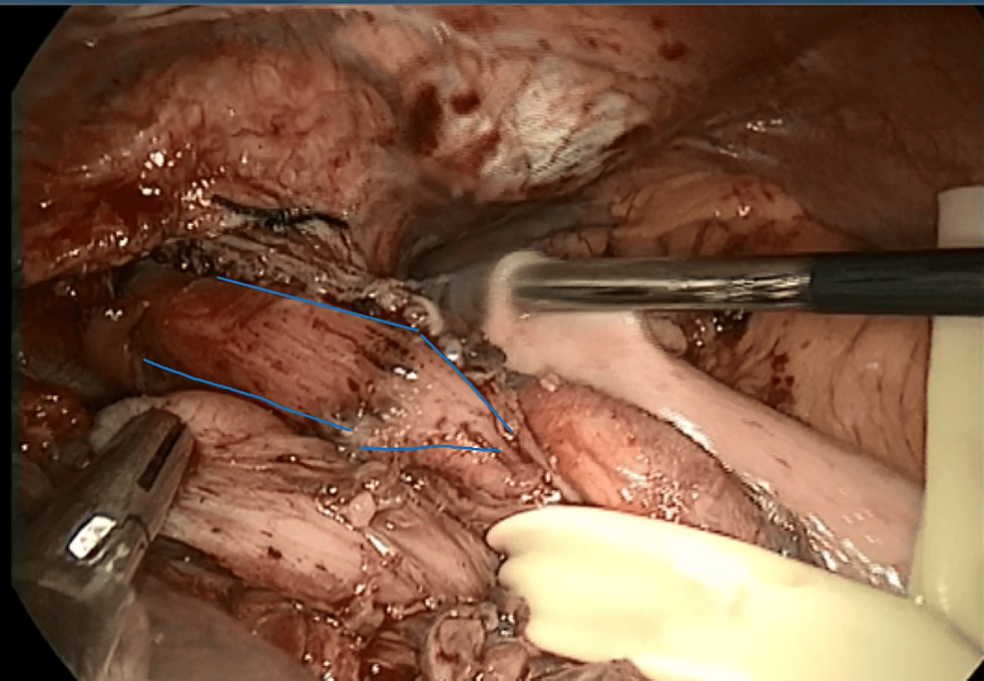
Preparation for Toupet fundoplication following a Heller myotomy, blue lines depict the cut edge of the esophageal muscles

**Figure 12:**
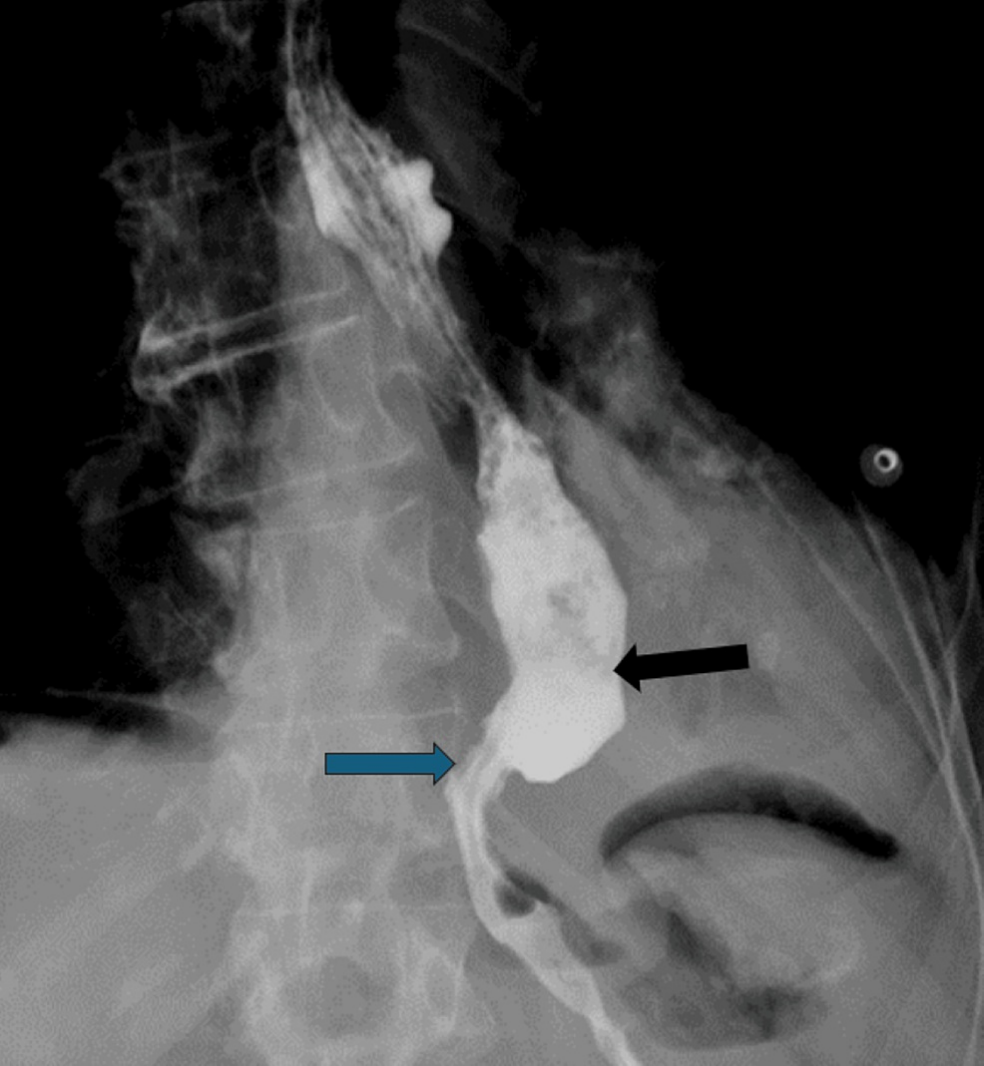
POD #1 esophagram showing decreased esophageal dilatation (black arrow) and flow through the gastroesophageal junction (blue arrow)

The left lobe liver was eventually pushed to the right upper quadrant with adjustment of the liver retractor, which allowed for proper exposure of the hiatus (Figure 10). A high mobilization of the esophagus was performed till we had about 4 cm of esophagus below the GE junction. Both the anterior and the posterior vagus nerves were clearly identified and kept out of harm's way. A portion of the gastroesophageal fat pad was dissected free to visualize the GE junction. We then proceeded to perform the myotomy. An area was chosen between the anterior and posterior vagus nerves and scored with a Bovie cautery. The longitudinal muscle fibers of the esophagus were separated with a Kittner till the circular muscle was visible. Each fiber was isolated with Maryland forceps and divided with a Harmonic scalpel. An 8 cm or longer myotomy was made going completely across the GE junction for about 2 cm. An EGD was performed that confirmed an adequate myotomy across the GE junction (Figure 11). Closure of the crus was done with a permanent silk suture. A posterior partial (Toupet) fundoplication was subsequently performed without a bougie. Intraoperative endoscopy revealed an adequate myotomy without any leaks. Post-operative esophagram showed improved transit through the GEJ, which was noted to be wide open (Figure 12). Her dysphagia resolved, and she was discharged on POD #2 with no post-operative complications. She was placed on two weeks of a full liquid diet without carbonation followed by one week of a soft diet and then she was advanced diet as tolerated. She was counseled that only part of the problem was fixed, i.e., the LES and that since there is no esophageal body peristalsis she need to take small bites of food and not chew too quickly. After nine months of FU, she has no dysphagia or reflux.

## Discussion

Achalasia is a relatively rare esophageal motility disorder with an incidence of 1.6/100,000 population [1]. Three types of achalasia have been described [2]. All types of achalasia demonstrate failed peristalsis in the esophagus and an increased LES pressure. Type I achalasia is also known as the classic type and has aperistalsis within the esophageal body and an increased LES pressure as measured by an IRP (>15 mm Hg) [3]. Type II has periods of pan esophageal pressurization found in greater than 20% of swallows on manometry. Type III is characterized by early or spastic distal contractions of the esophagus (>20%) [4]. Achalasia Quality of Life (ASQ) and Eckardt scores are self-reported scales to assess the severity of symptoms and include weight loss, dysphagia, retrosternal pain, and regurgitation. These scores can be used to compare symptoms pre- and post-intervention and monitor for treatment efficacy or failure [7]. PD is a good first step to provide symptomatic relief and may even offer similar therapeutic relief to LHM in some patients [8].^ ^Patients who have symptomatic recurrence after PD can be offered BTA or a myotomy, either a POEM or a Heller myotomy performed using minimally invasive techniques. The morbidity of both procedures is low. They both provide excellent relief of dysphagia, 92.7% after POEM and 90% after Heller at two years [9]. Post-operative GERD is noted to be higher in patients after POEM [9]. This includes symptomatic GERD, erosive esophagitis, and positive pH testing after the procedure. So, while a comparable relief of dysphagia is present with POEM, the incidence of gastroesophageal reflux with pathological consequences is higher. This is understandable as LHM adds an anti-reflux operation to the myotomy in the form of a partial fundoplication, which is not the case in a POEM. While both treatments are recommended for types 1 and 2, POEM is generally preferred for type 3 achalasia [10].^ ^This patient had minimal relief despite multiple PDs, and the options of LHM versus POEM were discussed. A multi-disciplinary team, which included gastroenterologists and radiologists, recommended proceeding with surgery. The recommendation was made due to the presence of a hiatal hernia as well as the altered anatomy due to dextrohepatica. Single-organ congenital lateralization defects are extremely rare within the literature and are limited to a few case reports and case series. A much rare condition is isolated organ situs inversus. In the foregut, most reports of isolated situs inversus are limited to isolated gastric situs inversus, dextrogastria. Most isolated liver malposition have described situs ambiguous, at the midline, usually associated with polysplenia [11-13].^ ^Our patient had the normal position of the foregut structures, including the stomach, spleen, pancreas, and duodenum, except for the isolated situs inversus of the liver.

## Conclusions

In this patient because of the unusual anatomical location of the liver, performing an LHM was quite challenging. The surgery was divided into three parts: 1) visualizing the hiatus, we discovered that it was easier and necessary to displace the larger left lobe of the liver toward the right upper abdomen rather than trying to lift it up on the left side; 2) performing the Heller myotomy, and 3) performing the posterior partial (Toupet) fundoplication. By displacing the larger left lobe of the liver in this situation, we were able to safely complete an LHM with adequate length and distally across the gastroesophageal junction. She had an uncomplicated post-operative course and at follow-up has continued to show improvements in her dysphagia and her quality of life.
